# PADAr: physician-oriented artificial intelligence-facilitating diagnosis aid for retinal diseases

**DOI:** 10.1117/1.JMI.9.4.044501

**Published:** 2022-07-25

**Authors:** Po-Kang Lin, Yu-Hsien Chiu, Chiu-Jung Huang, Chien-Yao Wang, Mei-Lien Pan, Da-Wei Wang, Hong-Yuan Mark Liao, Yong-Sheng Chen, Chieh-Hsiung Kuan, Shih-Yen Lin, Li-Fen Chen

**Affiliations:** a National Taiwan University, Graduate Institute of Biomedical Electronics and Bioinformatics, Taipei, Taiwan; b National Yang Ming Chiao Tung University, School of Medicine, Department of Ophthalmology, Taipei, Taiwan; c Taipei Veterans General Hospital, Department of Ophthalmology, Taipei, Taiwan; d National Yang Ming Chiao Tung University, Institute of Brain Science, College of Medicine, Taipei, Taiwan; e Academia Sinica, Institute of Information Science, Taipei, Taiwan; f Taipei Veterans General Hospital, Integrated Brain Research Unit, Department of Medical Research, Taipei, Taiwan; g National Yang Ming Chiao Tung University, Information Technology Service Center, Taipei, Taiwan; h National Yang Ming Chiao Tung University, Department of Computer Science, Hsinchu, Taiwan; i National Taiwan University, Department of Electrical Engineering, Taipei, Taiwan; j National Taiwan University, Gaduate Institute of Biomedical Electronics and Bioinformatics, Taipei, Taiwan

**Keywords:** computer-aided diagnosis, multi-retinopathy classification, lesion-sites visualization

## Abstract

**Purpose:**

Retinopathy screening via digital imaging is promising for early detection and timely treatment, and tracking retinopathic abnormality over time can help to reveal the risk of disease progression. We developed an innovative physician-oriented artificial intelligence-facilitating diagnosis aid system for retinal diseases for screening multiple retinopathies and monitoring the regions of potential abnormality over time.

**Approach:**

Our dataset contains 4908 fundus images from 304 eyes with image-level annotations, including diabetic retinopathy, age-related macular degeneration, cellophane maculopathy, pathological myopia, and healthy control (HC). The screening model utilized a VGG-based feature extractor and multiple-binary convolutional neural network-based classifiers. Images in time series were aligned via affine transforms estimated through speeded-up robust features. Heatmaps of retinopathy were generated from the feature extractor using gradient-weighted class activation mapping++, and individual candidate retinopathy sites were identified from the heatmaps using clustering algorithm. Nested cross-validation with a train-to-test split of 80% to 20% was used to evaluate the performance of the screening model.

**Results:**

Our screening model achieved 99% accuracy, 93% sensitivity, and 97% specificity in discriminating between patients with retinopathy and HCs. For discriminating between types of retinopathy, our model achieved an averaged performance of 80% accuracy, 78% sensitivity, 94% specificity, 79% *F*1-score, and Cohen’s kappa coefficient of 0.70. Moreover, visualization results were also shown to provide reasonable candidate sites of retinopathy.

**Conclusions:**

Our results demonstrated the capability of the proposed model for extracting diagnostic information of the abnormality and lesion locations, which allows clinicians to focus on patient-centered treatment and untangles the pathological plausibility hidden in deep learning models.

## 1 Introduction

Retinopathy is an important cause of visual impairment, which is generally irreversible in its later stages. The resulting presentation of drusen, cellophane, exudate, hemorrhage, or chorioretinal scarring can have a profound effect on the vision of its victims, in which the most common causes may be diabetic retinopathy (DR),^1^ age-related macular degeneration (AMD),^2^ cellophane maculopathy (CM),^3^ and pathological myopia (PM).^4^ The asymptomatic nature of retinopathy in the initial stages means that regular screening via digital imaging is promising for early detection and timely treatment.^5^

Color fundus imaging is a non-invasive cost-effective tool for ophthalmological examinations.^6^ A number of models based on convolutional neural networks (CNNs) have been developed to facilitate the classification of retinopathies based on color fundus images.^7^^–^^12^ One recent CNN-based study reported that salient regions obtained from gradient-weighted class activation mapping (Grad-CAM++)^13^ closely matched the regions identified by ophthalmologists.^14^ Retinopathic changes over time can be used to monitor disease progression and evaluate therapeutic outcomes.^15^ Clinical ophthalmologists rely heavily on digital imaging for diagnostics; however, manual tracking can be arduous and time-consuming. Clinicians require user-friendly computer-aided diagnostic tools to automate the process of identifying regions with retinopathic abnormalities, and to monitor changes in those areas over time in order to facilitate decision-making and thereby alleviate their workload.

**Fig. 1:**
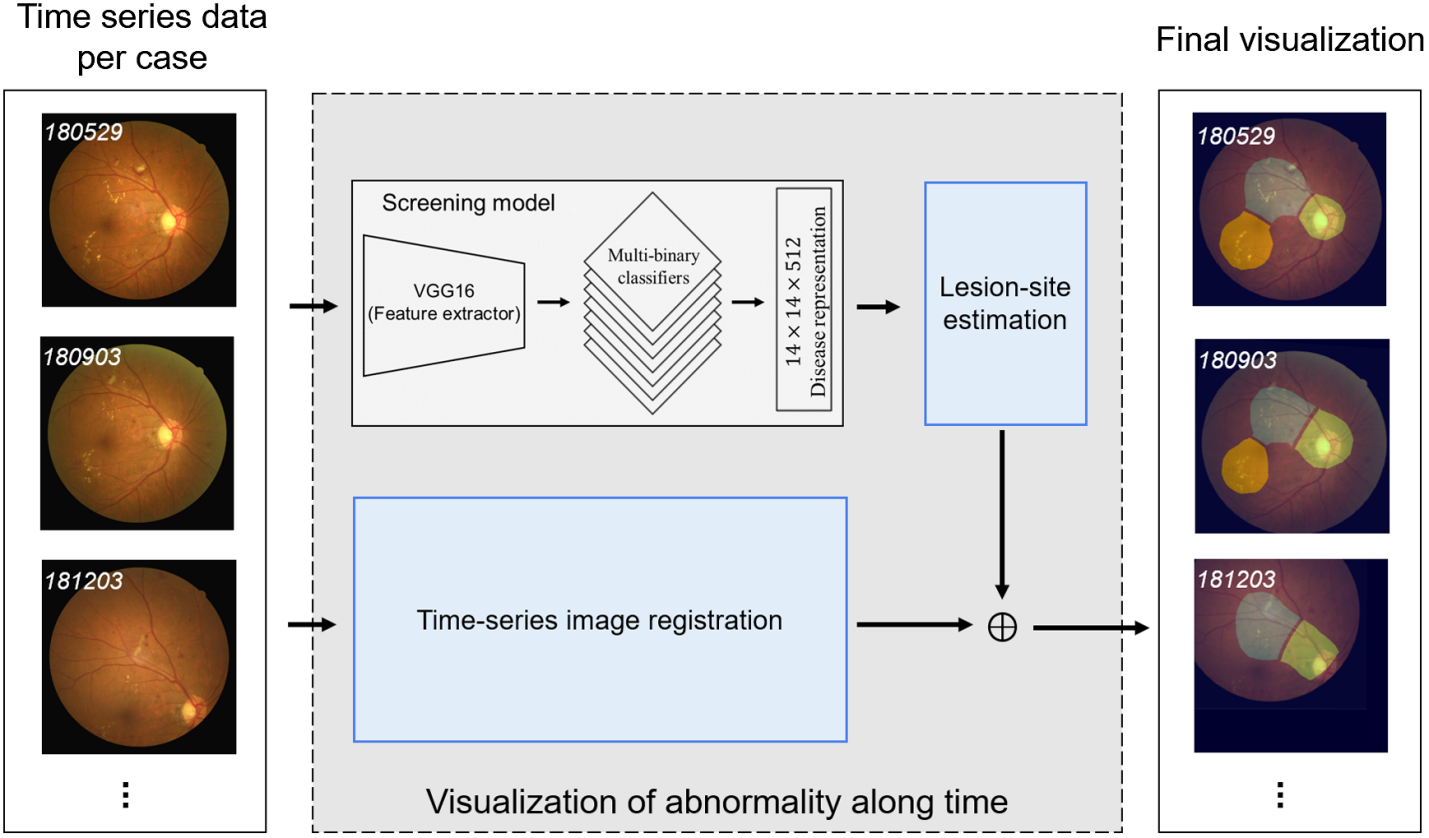
Overview of the PADA system.

In the current study, we developed an artificial intelligent (AI) diagnostics platform for screening multiple retinopathies and monitoring regions of potential abnormality over time. A schematic illustration of the proposed system, referred to as the physician-oriented AI-facilitating diagnosis aid system for retinal diseases (PADAr), is shown in Fig. 1. We employed machine learning techniques based on fundus images from 304 eyes affected by AMD, DR, CM, or PM, as well as healthy controls (HCs). It is worth noting that all training data had previously been labeled by a retina specialist (Dr. P.K. Lin). The proposed framework performs two fundamental operations: screening and monitoring. The screening model applies a shared-weight feature extractor to fundus images and then uses multiple-binary CNN-based classifiers to formulate outcome predictions. A corresponding heatmap was obtained from the last convolutional layer of the trained feature extractor using Grad-CAM++^13^ to highlight regions of potential abnormality, and thereby, differentiate HCs from cases requiring attention. In the second stage (i.e., monitoring model in Fig. 1 blue box), the heatmaps are registered over time using affine transforms estimated using a speeded-up robust features (SURF) descriptor^15^^–^^17^ based on the corresponding fundus image. We applied lesion-site estimation on each transformed heatmap to visualize change in retinopathic abnormalities over time. This study proposed a novel hybrid machine learning architecture by combining CNN, SURF descriptors, and clustering to automate the process of visualizing potential lesions over time. Our findings suggest that this type of algorithm could facilitate early diagnosis and the tracking of disease progression, contingent on the development of larger, more diverse datasets.

## 2 Materials and Methods

### 2.1 Data Acquisition and Preparation

This study was approved by the Ethics Committee of the Institutional Review Board of Taipei Veterans General Hospital, Taiwan (2018-08-003CC accepted November 26, 2018). Participants provided written informed consent allowing the retrospective collection of their retinal images. Participants were included if they were diagnosed with a major retinopathy in either eye, such as AMD, DR, CM, and PM. A total of 200 participants were selected for inclusion by a retina specialist from the Department of Ophthalmology of Taipei Veterans General Hospital in Taiwan. Sampling covered the period from 2002 to 2019.

Color fundus images of multiple fields were captured using multiple cameras equipped with lenses covering a field-of-view of 35 deg to 55 deg. The multiple fields were indicated using the seven fields designated in the general early treatment of diabetic retinopathy study (ETDRS) protocol: optic disc centered field (F1); macular centered field (F2); and all peripheral fields (F3, F4, F5, F6, and F7). Images lacking anatomical landmarks (e.g., optic disc, vessels, and macula) were removed. The images were cropped to 2201×2201 pixels. For visualization, all images were resized to 512×512 pixels. The 304 eyes (N=4908) included in the study were labeled as follows: HC (25 eyes, N=367), AMD (120 eyes, N=2029), DR (77 eyes, N=1681), CM (51 eyes, N=436), and PM (31 eyes, N=395). The dataset was divided into two subsets using an 80 to 20 split; that is, 80% of images were used as training validation data (N=4082) and 20% were used as test data (N=826). Participants who underwent more than two examinations (N=160) were selected to assess abnormalities over time.

### 2.2 Screening Model

**Fig. 2:**
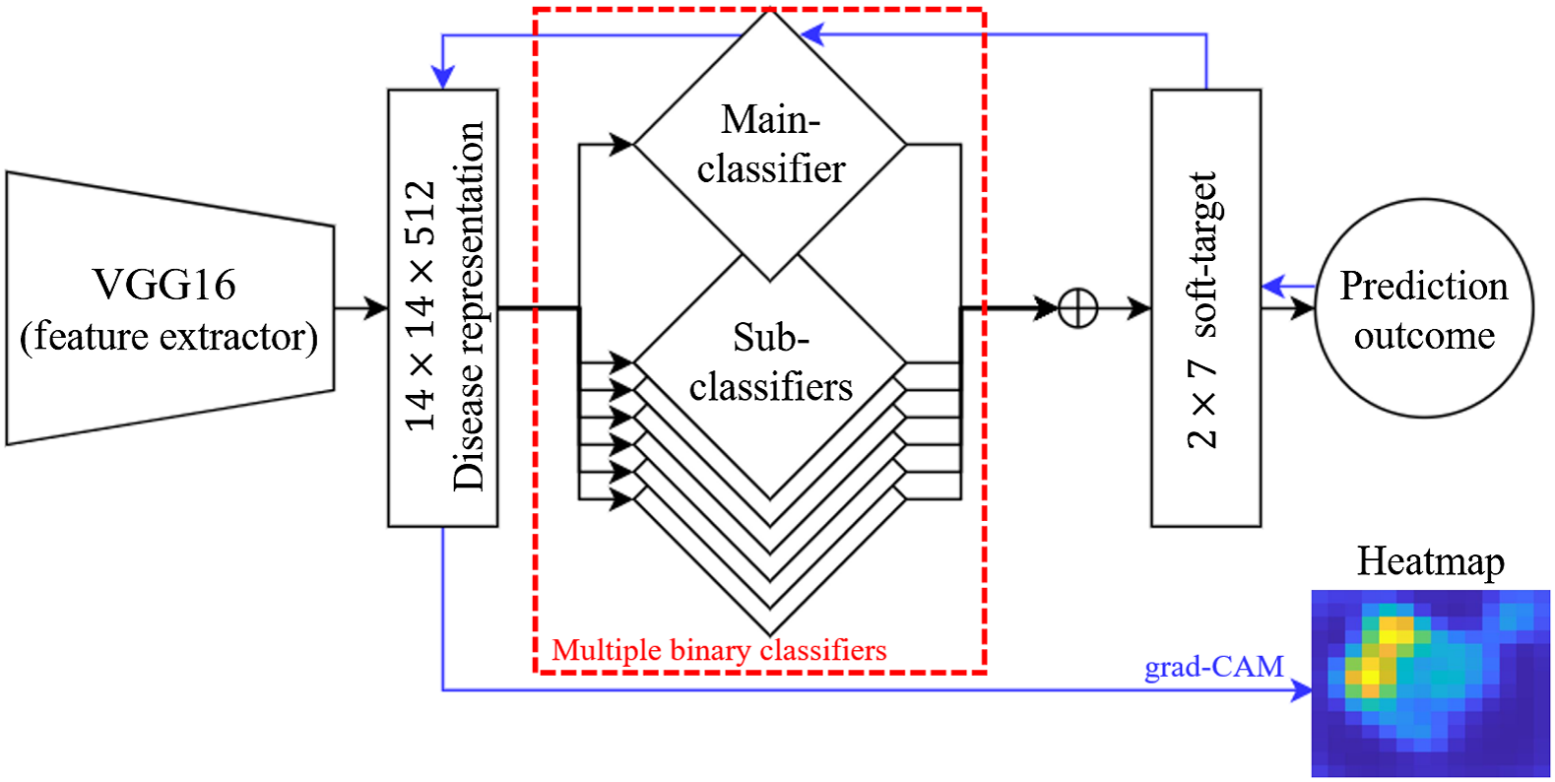
Architecture of proposed CNN-based screening model.

The present study proposed two models. The screening model (Fig. 2) based on multi-class classification employs a shared-weight feature extractor using VGG16^18^ as a backbone, a sub-network with multi-binary CNN-based classifiers for generating soft-target information, and a final fully connected (FC) layer for integrating the soft-target information to predict the class and generate the corresponding heatmap. The diseases representations (14×14×512) obtained from the last convolutional layer of a shared-weight feature extractor with global average pooling. We then removed the fully connected part of the VGG16 and employed multiple binary classifiers, including a main-classifier and the six sub-classifiers, providing soft-target information to the final FC layer. Each classifier contains three FC layers with the rectified linear unit function as activation, three dropout layers with a dropout rate of 0.2, and one softmax layer.

The main-classifier is used to discriminate between cases of retinopathy and HCs and six binary classifiers are used to differentiate cases between each pair of four types of retinopathy (AMD, DR, CM, and PM). The final FC layer integrates soft-target information obtained from all of the classifiers to predict the outcomes. We incorporated Grad-CAM++ to obtain the corresponding heatmap from the class of interest (i.e., retinopathy). Essentially, Grad-CAM++ generates the heatmap by a weighted combination of latent feature channels from the last convolutional layer. The weights for feature channels reflect their respective importance in prediction of a given class, which is estimated from the gradient of guided back-propagation. Grad-CAM++ is shown to achieve better localization compared to Grad-CAM^19^ by providing improved formulations for estimating the channel weights. Majority voting is used to determine the final prediction outcome for each patient.

Prior to model training, all input images were augmented by horizontal flipping, rotation [−36  deg,+36  deg], and translation of width and height [−10%,+10%] to resolve the problems of overfitting, small sample size, and an imbalance in available data for model training.^20^^,^^21^ The images (5000 in each class) were then resized to 224×224 pixels via bilinear interpolation for model training.

Training was implemented in three steps. We first replaced the last three fully connected layer of VGG16 with one binary main-classifier, utilized ImageNet^22^ pretrained weights to train the feature extractor from scratch and fine-tuned the network using our dataset to classify between cases of retinopathy and HCs. We then trained six binary sub-classifiers with the estimated weights of the feature extractor. Finally, we trained the final FC layer using soft-target information obtained from the trained classifiers including one binary main-classifier and six binary sub-classifiers. We used the binary cross-entropy loss function for training each binary classifier and utilized the categorical cross-entropy for training final screening model. For the hyper-parameters of all networks, we employed the Adam optimizer^23^ with an initial learning rate of $1\times 10^{-5}$, a final learning rate of $1\times 10^{-8}$, and batch size of 32. The learning rate decayed by a factor of ten over ten epochs showing no improvement in validation loss.

We performed 5×5-fold nested cross-validation (CV)^24^ to evaluate the performance of the feature extractor. No significant difference was observed among the folds from the feature extractor; therefore, we applied holdout CV for evaluating six binary sub-classifiers and the final FC layer. Model performance was measured in terms of accuracy, precision, sensitivity, specificity, F1-score, the area under curve (AUC) of receiver operating characteristic curve,^25^ and Cohen’s kappa coefficient.^26^ For each performance metric, macro-average was also calculated by the arithmetic mean of all individual classes. A retina specialist (P.K. Lin) also visually examined the candidate sites in the testing data for validating the efficacy of the proposed model.

### 2.3 Visualizing Abnormalities Over Time

The second model proposed in this work was used to monitor and visualize candidate lesion sites based on results from the aforementioned screening model at various time points for each patient. Time-series image registration was adopted to align images acquired from multiple time points from a single participant. For each image, control points were automatically extracted using SURF algorithm, and the time-series images and their corresponding heatmaps were registered to the reference image. Subsequently, a clustering algorithm was used to identify candidate sites based on their relevance to identified abnormalities.

#### 2.3.1 Time-series image registration

**Fig. 3:**
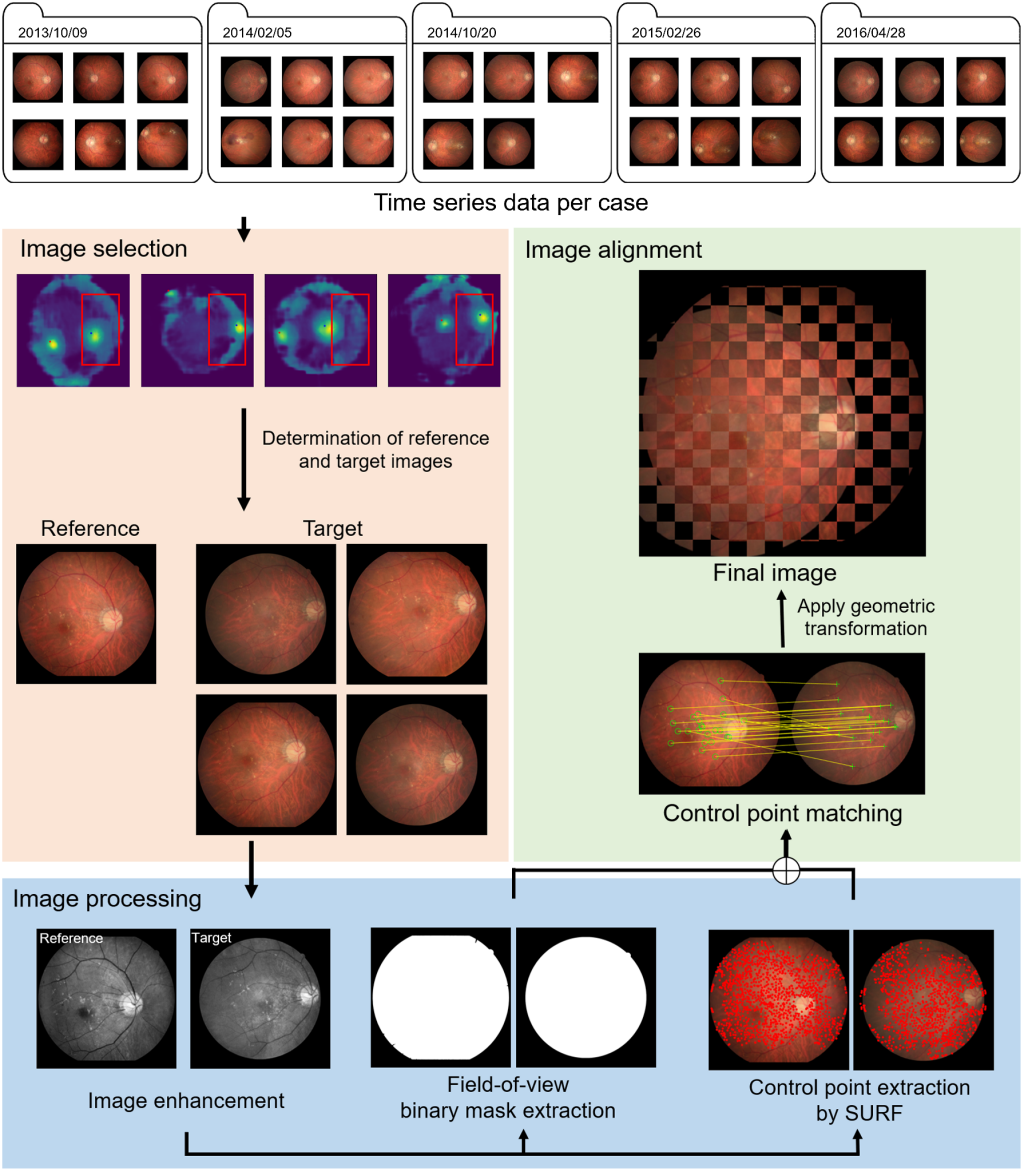
Illustration of proposed time-series image registration. The mosaic image indicates registration performance by combining reference and target images.

The schema of the proposed time-series image registration method is shown in Fig. 3, including image selection, control point extraction, and control point matching. For each image, we first detected the location of the optic disc $\left(X_{\mathrm{disc}},Y_{\mathrm{disc}}\right)$ using pixel-wise distance regression based optic disc detection approach.^27^ The region of interest (ROI) was defined as $\left(X_{\mathrm{ic}}\pm 0.3\times {\mathrm{Image}}_{\mathrm{width}},Y_{\mathrm{ic}}\pm 0.25\times {\mathrm{Image}}_{\mathrm{height}}\right)$, where ic refers to the image center. Images with the disc located within the ROI were selected as macula-center fundus images. For each patient, the macula-center image with the shortest distance between disc location and the center of ROI was then selected as a reference for registration.

Second, a green channel image extracted from each macula-center image was enhanced using the contrast limited adaptive histogram equalization filtering algorithm,^28^ whereupon the intensity was normalized to [0, 1] and resampled to 512×512 pixels. The field-of-view binary mask was derived using Otsu’s thresholding,^29^ followed by an erosion operator with 5 mm around the edge of the mask. Control points were extracted using the SURF algorithm.^15^^–^^17^

Third, the correspondence between control points $S_{X}$ in the reference image (X) and control points $S_{Y}$ in every macular-centered image (Y) was estimated using the efficient approximate nearest neighbor search,^30^ which computes the pairwise Euclidean distance between $S_{X}$ and $S_{Y}$. The affine transformation matrix of each $S_{X}$ and $S_{Y}$ pair was then estimated from the predicted correspondence and applied to the corresponding macular-center image using the robust m-estimator sample consensus algorithm.^31^ Finally, each candidate lesion site in the reference image was aligned to specific candidates in each of the transformed macular-center images (acquired at different time points) by calculating the shortest distance between the reference and the target candidates.

#### 2.3.2 Identifying candidate lesion sites

**Fig. 4:**
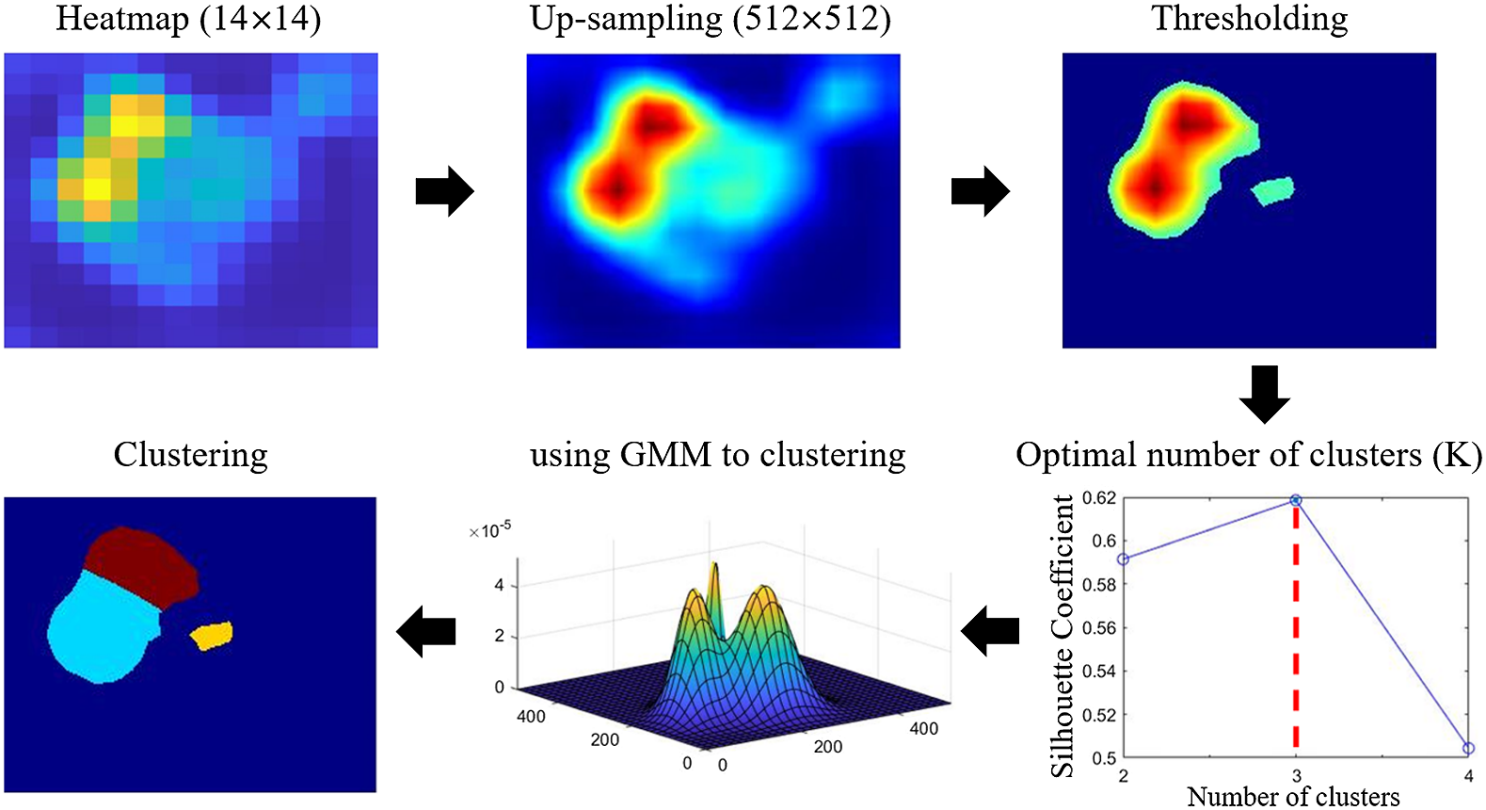
Pipeline of adaptive clustering algorithm. GMM: Gaussian mixture model.

An adaptive clustering algorithm was used to locate potential regions of abnormality (i.e., candidate lesion sites) on the heatmap derived from the screening model. The pipeline of our algorithm is shown in Fig. 4. The heatmap was first up-sampled to 512×512 pixels via bilinear interpolation. To visualize the abnormalities, we followed the standard procedure of Grad-CAM++^13^ and up-sampled the heatmaps to match the display image resolution (512×512 pixels) via bilinear interpolation. The intensity of the resulting heatmaps was then normalized to [0,1], followed by thresholding using the following Eq. (1):

Threshold=E(H)+σ, (1)

where E(H) and σ refer to the mean and standard deviation of heatmap H intensity, respectively. We then determined the optimal number of clusters (K) with maximum silhouette coefficient.^32^^,^^33^ Finally, we utilized a Gaussian mixture model^34^ to group pixels into clusters, each of which represented one candidate lesion site.

## 3 Results

### 3.1 Screening Performance

**Fig. 5:**
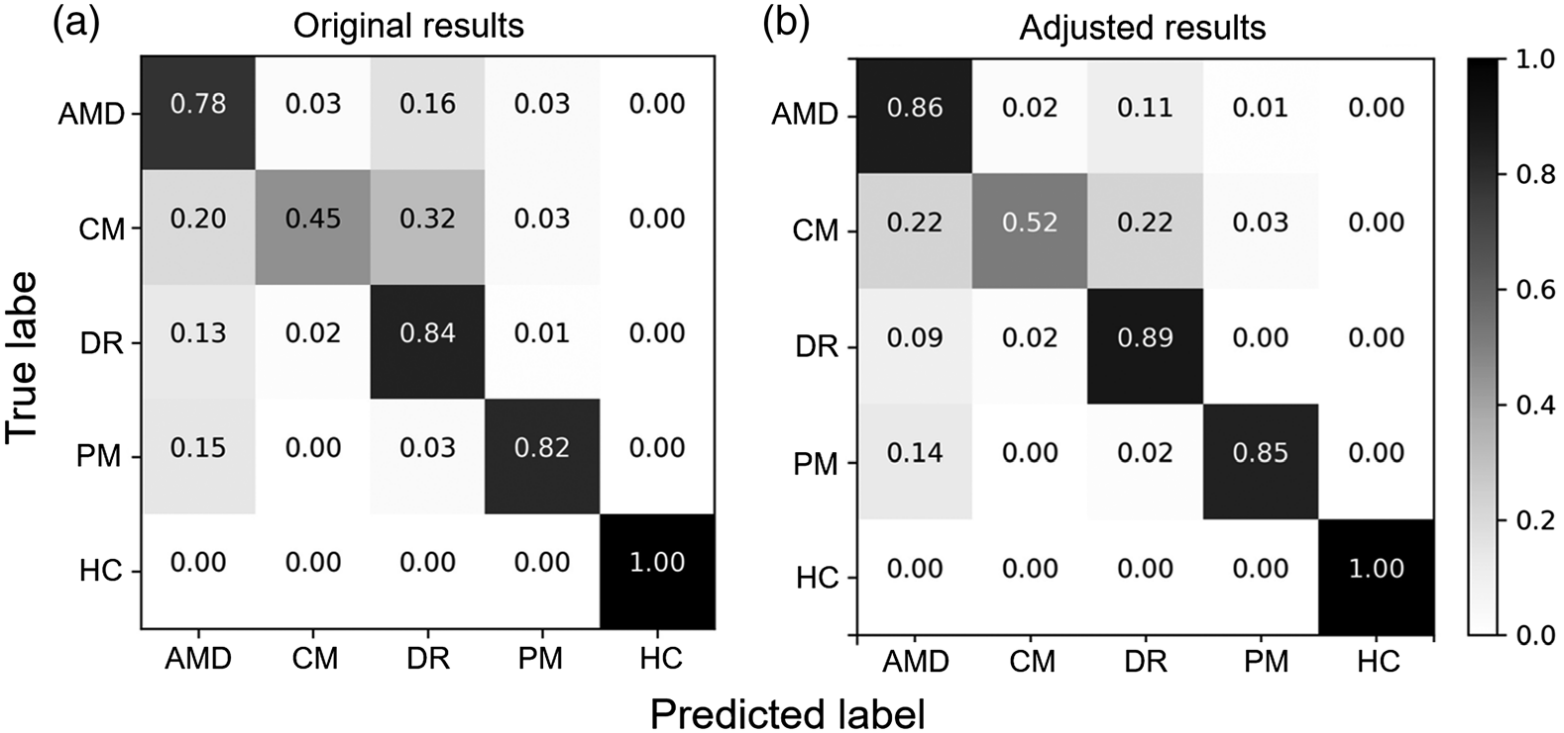
Confusion matrix of the screening model (a) before and (b) after removal of poor-quality images. AMD: age-related macular degeneration; DR: diabetic retinopathy; CM: cellophane maculopathy; PM: pathological myopia; and HC: health control.

In terms of screening, the proposed multi-binary-classifier model achieved macro-average accuracy of 0.80, precision of 0.81, sensitivity of 0.78, specificity of 0.94, F1 score of 0.79 (Table 1), AUC of 0.94, and Cohen’s kappa coefficient of 0.70. These results were obtained from uncleaned images captured during funduscopic examinations. A confusion matrix is presented in Fig. 5(a). As shown in Table 1 and Fig. 5(b), the removal of poor-quality images improved average accuracy by 5.4% and precision as follows: AMD (4%), DR (5%), CM (8%), and PM (10%).

**Table 1:** Performance of our proposed model. Numbers in parentheses indicate results based on recalculations following the removal of poor-quality images. AMD: age-related macular degeneration; DR: diabetic retinopathy; CM: cellophane maculopathy; PM: pathological myopia; and HC: healthy control.

	Precision	Sensitivity	Specificity	F1-score	AUC	Cohen’s Kappa
AMD	0.79 (0.83)	0.78 (0.86)	0.87 (0.90)	0.79 (0.85)	0.89	—
DR	0.78 (0.86)	0.84 (0.89)	0.85 (0.90)	0.81 (0.87)	0.92	—
CM	0.68 (0.73)	0.45 (0.52)	0.98 (0.98)	0.55 (0.61)	0.90	—
PM	0.79 (0.89)	0.82 (0.85)	0.98 (0.99)	0.81 (0.87)	0.98	—
HC	1.00 (1.00)	1.00 (1.00)	1.00 (1.00)	1.00 (1.00)	1.00	—
Macro average	0.81 (0.86)	0.78 (0.85)	0.94 (0.96)	0.79 (0.85)	0.939	0.701 (0.785)

The performance of the main classifier was assessed using repeated nested CV with an outer five-fold CV and an inner five-fold CV. The main-classifier for discriminating between patients and HCs achieved high mean-macro-average accuracy of 0.99±0.003 (precision, 0.99±0.005; recall, 0.93±0.017; *F*1 score, 0.96±0.012; and Cohen’s kappa coefficient, 0.91±0.023).

### 3.2 Candidate Regions of Disease

**Fig. 6:**
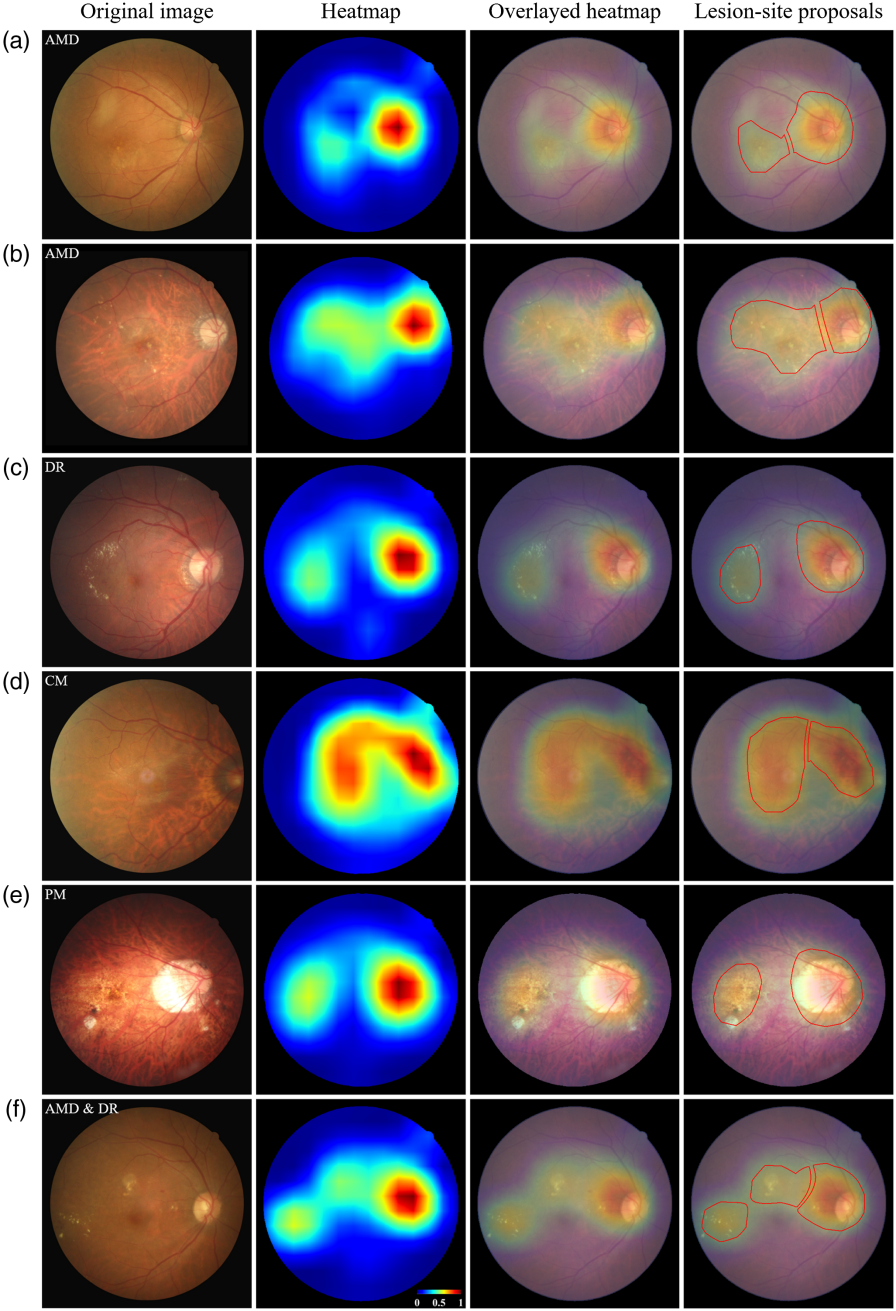
Heatmaps of five retinopathy images generated from screening model: (a) dry-type AMD; (b) wet-type AMD; (c) DR; (d) CM; (e) PM; and (f) both AMD and DR. The original fundus images and corresponding heatmaps are respectively presented in the first and third columns. The second column displays the original images overlaid with their corresponding heatmaps. The fourth column displays the original images overlaid with their corresponding heatmap and candidate lesion-sites (in red), highlighting potential regions of abnormality. AMD: age-related macular degeneration; DR: diabetic retinopathy; CM: cellophane maculopathy; and PM: pathological myopia.

The proposed model provides diagnostic information from heatmaps pertaining to the retina for use in identifying candidate locations of disease. Figures 6(a) and 6(b) show images showing examples of regional retinopathy in the AMD, including drusen and edema. Figure 6(c) illustrates instances of hemorrhage and exudate in a case of DR. The heatmap of CM in Fig. 6(d) focuses on the optic disc extending to the macula. The heatmap of PM in Fig. 6(e) focuses on the crescent near the disc and macular degeneration. Figure 6(f) highlights drusen and exudate. Note that most of the heatmaps highlighted the optic disk.

**Fig. 7:**
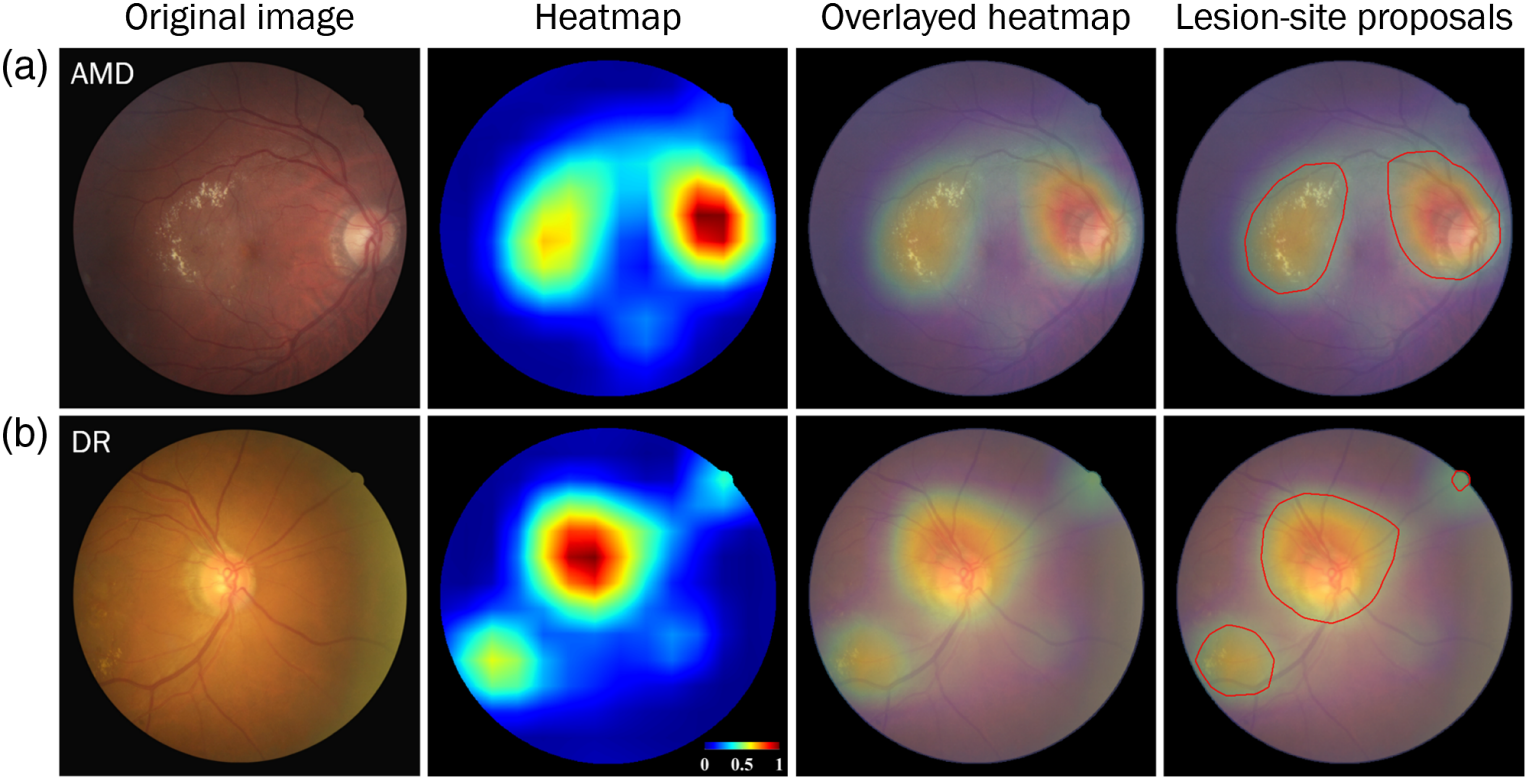
Heatmaps of misclassified cases. (a) AMD case misclassified as DR and (b) DR misclassified as AMD (AMD: age-related macular degeneration; DR: diabetic retinopathy).

Figure 7 shows two examples of prediction error, in which an image of the AMD was misclassified as DR [Fig. 7(a)] and an image of the DR was misclassified as AMD [Fig. 7(b)]. Regardless, the heatmaps provided reasonable candidate sites of retinopathy, including sites around the macula and optic disc (third column in the panel).

### 3.3 Visualizing Candidate Regions of Abnormality Over Time

**Fig. 8:**
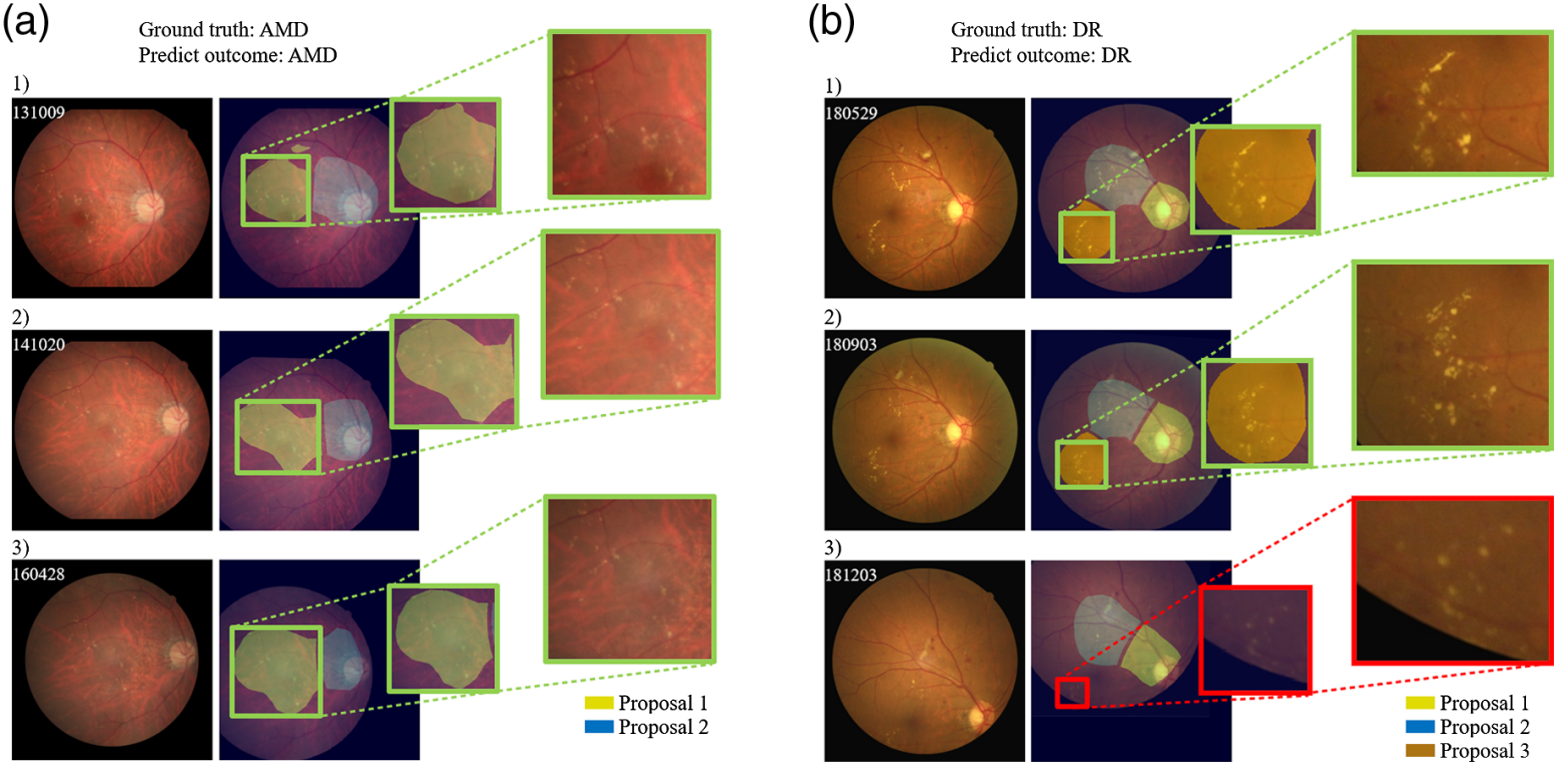
Visualization results from two fundus images obtained at different timepoints, where (1), (2), and (3) denote the first-, second-, and third-time scans, respectively. (a) and (b) the left column displays the original color fundus images. The middle column displays lesion-site candidates over time. The right column displays close-up images from one of the lesion-site candidates indicating a potential region of abnormality. It is worth noting that color is used to differentiate specific candidates over time. Green bounding boxes indicate correctly identified regions, whereas the red bounding boxes denote the miss-detection regions.

Figure 8 shows two cases illustrating changes in retinopathy along time. In Fig. 8(a), the proposed system highlighted candidate retinopathic abnormalities in the AMD (e.g., drusen), in which the condition remained stable in subsequent yearly follow-up examinations. In Fig. 8(b), the system highlighted the progress of exudate and hemorrhage in the DR in monthly follow-up examinations, in which the severity of the conditions gradually decreased. These results demonstrate the effectiveness of the system in tracking retinopathies via funduscopic examination.

## 4 Discussion

Locating abnormalities in the retina is crucial to diagnostic decision-making. Previous studies have reported that heatmaps obtained from Grad-CAM++ can be used to highlight such abnormalities in instances of single retinopathy (e.g., AMD or DR).^14^^,^^35^^,^^36^ Note however that many patients suffer more than one retinopathy in either or both eyes; therefore, we proposed the use of a main-classifier to differentiate patients from HCs in order to detect all potential abnormalities within the regions identified by the retina specialist (Dr. P.K. Lin). As shown in Figs. 6 and 7, the resulting heatmap was able to locate all potential regions of abnormality, regardless of whether the prediction outcome was correct. Our weakly supervised approach to learning pixel-wise labeling directly from image-level annotation is meant to reduce the effort required to label ground-truth locations of retinopathy. Experiments demonstrated the feasibility and efficacy of the proposed method in locating potential sites of retinopathic abnormality.

Our model also demonstrated competitive classification performance when compared to the other retinopathy detection models in the literature, either in distinguishing retinopathy from HCs or discriminating between types of retinopathy. Table 2 gives the reported performance of other binary classification models. Compared to other binary retinopathy classification models, the proposed system demonstrated superior sensitivity compared to other models. It is worth noting that most binary classification models in the literature only involve detecting a single type of retinopathy. In contrast, the binary classification in our study involves distinguishing four types of retinopathy from HCs. With larger diversity in the disease characteristics, it is thereby a more difficult task compared to detecting a single disease type. Nonetheless, although studies by Gulshan et al.^37^ and Zhang et al.^38^ reported higher AUC and F1 score, respectively, the proposed model still achieved competitive performance in most respect under significant larger disease diversity.

**Table 2:** The reported performance of binary retinopathy classification models in the reviewed literature, compared with the proposed system. The best performance according to each metric is highlighted by boldface.

	Database	Classification	AUC	Accuracy	Sensitivity	Specificity	F1 score
Gargeya and Leng^35^	Private dataset	DR	0.97	—	0.94	**0.98**	—
	E-Ophtha	DR	0.95	—	0.9	0.94	—
Choi et al.^8^	STARE	Nine diseases	0.903	—	0.803	0.855	—
Tan et al.^10^	Private dataset	AMD	—	0.9545	0.9643	0.9375	—
Gulshan et al.^37^	Private dataset	DR	**0.98**	—	0.921	0.952	—
Zhang et al.^38^	Private dataset	DR	—	0.98	**0.98**	—	**0.98**
Zago et al.^39^	Messidor	DR	0.912	—	0.94	—	—
Das et al.^40^	DIARETDB1 (train)	DR	—	0.974	0.976	0.972	—
	Private dataset (test)						
Proposed	Private dataset	Four diseases	0.939	**0.99**	**0.98**	0.97	0.85

Table 3 shows the reported performance in the literature for distinguishing between multiple retinopathy types. Compared to other models, the proposed system demonstrated superior sensitivity. It is worth noting that the classification of CM yielded a lower sensitivity compared to other types of retinopathy. As previously shown in Fig. 5, CM is occasionally confused with other types of retinopathy, such as AMD and DR. We hypothesize that this lower performance is attributable to the confounding effects by the prevalence of myopia in Taiwan. Nonetheless, the proposed system still serves as an effective screening tool with its ability to accurately detect the presence of retinopathy, despite occasional confusion between retinopathy types. In-depth examinations using other imaging techniques (such as optical coherence tomography and fluorescein angiography) can be used after the screening stage for a more accurate diagnosis of retinopathy types.

**Table 3:** The reported performance of multi-class retinopathy classification models in the reviewed literature, compared with the proposed system. ARIA: automated retinal image analysis database; STARE: structured analysis of the retina database; ODIR: ocular disease intelligent recognition database.

	Database	Model	Classification	AUC	Accuracy	Sensitivity	Specificity	F1-score	Kappa
Arunkumar, et al.^12^	ARIA	Dimension reduced deep learning	Three classes (AMD/DR/normal)	—	0.9673	0.7932	0.9673	—	—
Choi, et al.^8^	STARE	VGG19 random forest	10 classes (normal, BDR, PDR, Dry AMD, Wet AMD, RVO, RAO, hypertensive retinopathy, coat’s disease, and retinitis) three classes (normal, BDR, and dry AMD)	—	0.305	—	—	—	0.224
				—	0.728	—	—	—	0.577
Gour, et al.^41^	ODIR	VGG16-SGD	Eight classes (normal, diabetes, glaucoma, catareact, AMD, hypertension, myopia, and other)	0.6888	0.8906	—	—	0.8557	—
			Normal	-	0.66	0.77	0.21	—	—
			Glaucoma		0.67	0.4	0.6		
			Diabetic retinopathy		0.93	0.05	0.94		
			AMD		0.94	0.06	0.93		
			Hypertension		0.95	0	0.99		
			Cataract		0.96	0	1		
			Myopia		0.94	0.11	0.94		
			Other abnormalities		0.73	0.74	0.32		
Rajan, et al.^42^	STARE	CNN	10 classes (normal, BDR, PDR, Dry AMD, Wet AMD, RVO, RAO, hypertensive retinopathy, coat’s disease, and retinitis)	—	0.42	—	—	—	—
Proposed	private dataset	VGG16-based	Five classes (normal, AMD, DR, PM, and CM)	0.93	0.86	0.85	0.94	0.79	0.7
			Normal	1	1	1	1	1	—
			DR	0.91	0.89	0.84	0.85	0.81	
			AMD	0.89	0.86	0.78	0.87	0.79	
			PM	0.97	0.85	0.82	0.98	0.81	
			CM	0.90	0.52	0.45	0.98	0.55	

It is worth noting that our study incorporated real-world data with minimal data cleaning and annotations. In the literature, screening models trained in real-world clinical settings are generally outperformed by those trained in a laboratory setting with carefully selected data,^43^^,^^44^ due to noise or artifacts originated from sub-optimal imaging equipment, patient movement, or exposure error.^45^^,^^46^ Nevertheless, our comparison results demonstrate that the proposed model achieved comparable performance to models trained with carefully selected data. Additionally, the proposed system infers location information from eye-based annotation in a weakly supervised manner, by which we sought to preserve the subclinical features of fundus images and to mitigate the labor-intensive annotation process. How to improve the detection performance and localization ability under the real-world data paradigm will be one of our future focus.

Monitoring disease progression from multiple examinations performed on different days provides quantitative and qualitative information by which to monitor disease progression. This process is critical to ensuring timely treatment; however, the process is time-consuming. Recent studies have reported that the discrimination of disease stage can help to reveal the risk of disease progression, particularly in areas such as the AMD and DR.^47^^,^^48^ Sequential changes in retinopathic characteristics observed in fundus images can be used to detail the evolution of retinopathy progression. In the current study, we developed a novel user-friendly tool by which to obtain assessments tailored to the individual for use in pinpointing the location of abnormalities from a single fundus image and visualizing changes in the corresponding disease spot region over time.

To the best of our knowledge, this is the first attempt to automate the location and visualization of retinopathic regions in the temporal domain. Our results demonstrates the capability of the proposed PADAr to identify potential retinopathy sites and perform longitudinal follow-ups of disease progression, suggesting its feasibility for facilitating clinicians in their decision-making process and focusing on patient-centered treatment.

## Code availability

The code to develop the screening model is based on Keras using TensorFlow as backend. Custom code was specific to our computing infrastructure and mainly used for data input/output and parallelization across computers.
